# 

**Published:** 1923-03

**Authors:** 


### PUBLIC HEALTH IN PARLIAMENT.

The following answers were given by Sir William Joynson-Hicks, for the Minister of Health.

### HOUSING.

Housing Statistics.—To Mr. Trevelyan: The number of houses erected or in course of erection under the State Assisted Scheme and under the Private Builders' Grant is as follows :—

| | |
|---|---|
| By local authorities and public utility societies | 176,000 |
| By private builders | 39,161 |
| By conversion of huts and hostels | 3,056 |
| | 218,217 |

State-aided Schemes.—To Mr. Trevelyan Thomson, who asked how many houses local authorities had been authorised to build under the late Government's assisted schemes: The scheme mentioned by the Hon. Member was limited to 176,000; these houses have now been allocated and there is not at present any margin available for redistribution.

Non-Subsidised Houses.—To Sir Kingsley Wood: My Rt. Hon. Friend has no general information as to the number of houses erected or being erected by private builders without the aid of a Government subsidy. So far as working class houses are concerned, he is aware that certain employers of labour are undertaking schemes; for example, a group of colliery proprietors has commenced a programme of 10,000 houses.

### MILK (SPECIAL) DESIGNATIONS ORDER.

Pasteurised Milk.—To Colonel Sir Arthur Holbrook: My Rt. Hon. Friend, the Minister of Health, will be pleased to consider any representations for the revision of the Order referred to. But he would like to remind the Hon. and Gallant Member that the object of the provisions in relation to pasteurised milk is to secure that the milk sold under this designation shall be as free as possible from infection and contamination; and there is a danger that any modification of those provisions would defeat this object.

### NATIONAL HEALTH INSURANCE.

Excessive Sickness Claims.—To Mr. R. Davies, who asked a question about Section 63 of the National Health Insurance Act, 1911, as amended by Section 38 of The National Health Insurance Act, 1918: No information is available as to the number of cases in which Approved Societies or Insurance Committees have made representations to persons or authorities that there has been excessive sickness amongst insured persons by reason of local conditions for which such persons or authorities are responsible. In the few cases in which representations have been made to the Ministry of Health as to excessive expenditure on sickness benefit in various localities, the allegations by the Society have been of a general character and have not been supported by actual figures of expenditure.

Finance.—To Mr. Sexton, who asked the amount annually paid from all sources under the National Health Insurance Act for any year since 1920, or including 1920, and the amount disbursed in sick benefit and administration, separately: For the year 1921, as regards England and Wales, the amounts are as follows :—

| | |
|---|---|
| Amount paid from all sources under the National Health Insurance Acts | £36,356,000 |
| Amount disbursed for benefits (including £7,200,000 sickness benefit) | £21,562,000 |
| Amount disbursed in administration | £4,827,000 |

### VOLUNTARY HOSPITALS.

Hospital Accommodation.—To Mr. Rhys Davies, who asked the number of persons at any recent date in England, Scotland and Wales who had failed to secure admission to hospitals for purposes of surgical operations; and whether he will also state the attitude of the Ministry towards the lack of accommodation in these institutions: As regards the first part of the question there is no information available. The second part raises issues which cannot conveniently be dealt with in reply to a question, but the Hon. Member will find the subject of hospital accommodation discussed in the Interim Report of the Voluntary Hospitals Commission.

### RECENT APPOINTMENTS.

### Medical Officers, Chaplains, etc.

Alderton, W. H., Chester; M.O.H., Lexden and Winstree Rural District Council.

Evans, Olwen, Medical Officer, Festiniog.

Fiddian, Dr., M.O. for Letchworth.

Gerrard, Dora Williams, M.B., Ch.B., D.P.H., Clinical Assistant Eye Infirmary, Edinburgh; Assistant M.O. Dundee. £450. (Edinburgh University.)

Graham, David James, Lieut.-Colonel, O.B.E., M.D., C.M., F.R.C.P.E., R.A.M.C.(T.A.); Surgeon Apothecary to His Majesty s Household at Holyrood Palace.

Hadden, Marie, Assistant Medical Officer of Health for Salford.

Mitchell, A. C., Herne Hill; Resident M.O., Borough Hospital, Croydon.

Robertson, William, M.D., D.P.H., Deputy M.O.H., Edinburgh; M.O.H., Edinburgh, £1,250 (Glasgow University).

Starling, E. H., F.R.S.; First Foulerton Professor.

Towers, A. F., M.D., D.P.H., Assistant Medical Officer, Wallsend; Medical Officer of Health, School Medical Officer and Medical Superintendent of Isolation Hospital, Jarrow (Edinburgh University).

Vines, H. W. C., Fellow of Christ's College, Cambridge; Foulerton Research Student.

### Public Health and Hospital Appointments.

Barnicolt, M., Foster Mother, Children's Homes, Steyning Union.

Cawley, Margaret, C.M.B., District Nurse, Earlestown, Lancashire; Health Visitor, Warrington (Wigan General Infirmary).

Crouch, C. J., a Sanitary Inspector under the Woolwich Borough Council.

Evans, H. W., Windsor; Chief General Attendant, Poor Law Institution, Hendon Union.

Evans, M. E., Wantage, Chief General Attendant, Poor Law Institution, Hendon Union.

Fagelman, T.; Health Visitor, Leeds (Holgate Hospital, Middlesbrough).

Gribble, Mary G.; Health Visitor and School Nurse, Oxford (St. Marylebone Infirmary).

Hart, M., Foster Mother, Children's Homes, Steyning Union.

Isherwood, Arthur, Sanitary Inspector, Bury St. Edmunds; Chief Sanitary Inspector for the Borough and Port Sanitary District of Lowestoft.

Lambert, S. Jane; Assistant County Superintendent and Inspector of Midwives, Cornwall County; County Superintendent Nurse, Isle of Wight (London Hospital).

Payne, Janet, almoner at the Radcliffe Infirmary, Oxford.

Ronaldson, Miss, almoner at the Elizabeth Garrett Anderson Hospital, Euston Road, N.W.

### Preston Hall Training Colony.

The Minister of Health, in a circular letter to County and County Borough Councils, Tuberculosis Joint Committees, and Metropolitan Borough Councils, states that it has been decided to utilise 100 places provided at the Preston Hall Sanatorium and Training Colony, Aylesford, Kent, by the Industrial Settlements (Incorporated) for Disabled Soldiers and Sailors. The courses of training at Preston Hall comprise market gardening, poultry and pig-keeping, &c. (70 places), and rural carpentry (30 places). Cases for vocational training are selected by the Ministry of Pensions, and the men are entitled to remain in the institution until completion of a special course of vocational training, the duration of which is not to exceed twelve months.

